# Commercially Pure Titanium Implants With Selenium and Hyaluronic Acid Coating for Dental Applications

**DOI:** 10.7759/cureus.52984

**Published:** 2024-01-26

**Authors:** Soorya Ganesh, Gheena S, Kalaiyarasan Madhu

**Affiliations:** 1 Oral Pathology, Saveetha Dental College and Hospitals, Saveetha Institute of Medical and Technical Sciences, Chennai, IND

**Keywords:** titanium, sol-gel coating, antibacterial effect, hyaluronic acid, selenium

## Abstract

Background

The current study demonstrated improved efficiency of commercially pure titanium (CP-Ti) dental implants with a biocompatible coating of selenium (Se) and hyaluronic acid (HA).

Methods

The sol-gel solution was made with hyaluronic acid and selenium nitrate in a 1:1 ratio. The coating sample was subjected to characterization studies such as Fourier Transform Infrared (FTIR) spectroscopy, surface morphology analysis, contact angle, etc., to confirm the coating. The antimicrobial activity of the coating was studied by analyzing the zone of inhibition.

Results

The FTIR spectroscopy was analyzed to confirm the functional groups of Se/HA. The surface morphology of the coated sample appeared as tiny needles with a plate-like surface. The potentiodynamic polarization studies revealed that the coated Ti samples exhibit superior corrosion resistance. The antimicrobial activity of *Staphylococcus aureus* was found to be more effective at a concentration of 100 mg. Furthermore, the basic criteria of biomaterials were analyzed for hemocompatibility studies, which suggested a non-hemolytic character.

Conclusion

The biocompatible coating of Se and HA has proven to be of superior corrosion resistance and antimicrobial activity resistance, which validate the usage of dental implants.

## Introduction

The main components of native bone are cells, collagen (a fibrous protein), hydroxyapatite, and water. It also has a nanocomposite structure that serves as the body's skeleton [1]. Bones are inflexible structures attaining and modifying shape according to their function. Although the neurovascular, endocrine, and vascular systems all play a major role in regulating bone metabolism, more research is needed to understand the complicated machinery of the bone’s reaction to internal and external stimulation [2]. Recently, metallic biomaterial implants have been gaining more importance based on implant applications. At present, various metallic biomaterials like cobalt, chromium, stainless steel, and titanium and their related alloys are employed for implant applications [3]. Among these materials, titanium (Ti) is an excellent metal widely employed in various industries, including dentistry, aerospace, medicine, etc. [4]. In the context of dental implants, Ti and Ti-based alloys are the most commonly employed materials due to their excellent properties and biocompatibility [5]. Ti exhibits excellent biocompatibility and is well-tolerated by the human body. When a Ti implant is placed in the jawbone, it undergoes osseointegration where the bone fuses with the implant surface, creating a stable and durable connection. Ti has a high strength-to-weight ratio, making it a strong and adaptable material. This property is crucial for dental implants as they need to withstand the forces exerted during biting and chewing. When exposed to oxygen, Ti produces a thin oxide layer on its surface that offers remarkable corrosion resistance. This oxide layer protects Ti from degradation in the oral environment [6]. Ti has a low thermal conductivity compared to other metallic materials. This characteristic can be advantageous in dental implants as it diminishes the risk of temperature sensitivity and discomfort for the patient. Ti is non-magnetic, which is beneficial in certain medical and dental applications, including magnetic resonance imaging (MRI) and other scanning examinations [7]. Non-magnetic properties ensure that the Ti implant does not interfere with imaging procedures.

However, the major problem of implant failure is bio-inertness and biofilm formation on the implant surface. This problem was improved by using selenium (Se) and hyaluronic acid (HA) against the biofilm formation of the implant surface. Se is an important trace component; it regulates redox processes and antioxidant activity [8]. Se deposited in the Ti implant can induce osteoblast activity and osteogenic differentiation properties [8]. Zhou J *et al.* have studied different concentrations of Se (3-14 wt%). The optimized 8 wt% exhibits good antibacterial activity, and it helps to increase the osteogenic activity [9].

HA is a naturally occurring substance found in the human body and has been used in various medical and dental applications, including tissue engineering and wound healing [10]. It has been investigated for its potential benefits in promoting tissue regeneration and reducing inflammation. It has been studied for its potential therapeutic effects in various diseases and conditions. The present study is a successful attempt at addressing the lacunae of limited scientific literature available on the specific combination of Se/HA-coated Ti for dental implants. Fabricated coated material is resistant to bacterial infection on the dental implant surface. The material can be potentially used for long-term implant applications.

## Materials and methods

Sample preparation

Commercially pure titanium (CP-Ti) samples were purchased from Ti Anode India Pvt. Ltd., Chennai. The samples were cut into sizes of 1.5 cm x 1.5 cm x 2 mm thickness and washed with acetone and distilled water for 20 minutes using an ultrasonicator. The samples were treated with Kroll’s reagent for 5 seconds to remove any undesirable particles on the surface. After washing with distilled water, the samples were allowed to dry at room temperature before being utilized for coating. 0.5 g selenium nitrate (Se(NO_3_)_2_) was dissolved in 50 ml of condensed water and continuously stirred for 10 minutes. Then, 0.5 g of sodium hyaluronate was taken and dissolved in 50 ml distilled water for 10 minutes; this solution was added drop by drop in selenite solution under continuous slow stirring for 1 hour to obtain a homogenous solution. The prepared titanium sample was coated followed by dip coating. 

Characterization studies

Surface Morphology Studies

The surface topography of the coated sample was analyzed by using a Field Emission Scanning Electron Microscope (FE-SEM) with a JEOL Model (JSM-IT800 NANO SEM; Tokyo, Japan) instrument combined with an Energy Dispersive X-ray Spectrometer analyzer to find out the elemental composition. 

Fourier Transform Infrared (FTIR) Spectroscopy Studies

The presence of functional groups was identified by the Alpha II Bruker model spectrometer (Germany) connected to a personal computer and the coated sample was examined over the range of 4000 to 400 cm^-1 ^wave numbers.

Contact Angle

The water contact angle was determined for bare and coated samples using Model Phoenix 300 Plus (M/s Surface Electro Optics, South Korea) with a drop of water volume of 8 µl.

Electrochemical Studies

The bare and coated samples were evaluated for bio-corrosion using the potentiodynamic polarization method. The potentiodynamic polarization was examined by an electrochemical cell comprising a three-electrode system: reference electrode as calomel electrode, counter electrode as platinum, and working electrode as the coated sample that was exposed 1 cm^-1^ in simulated body fluid (SBF) solution. The experiment was carried out concerning open circuit potential (OCP) at a scan rate of 1 mVs^-1^ and polarization resistance was evaluated through the Stern-Geary equation as given below [11].

R_p_=(β_a_ x β_c_)/(2.3 i_corr_(β_a_ x β_c_))------(1)

Where β_a_ and β_c_ are the anodic and cathode slopes of the Tafel plot, R_p_ is polarization resistance, and i_corr_ is corrosion current density.

Antibacterial Activity

The coated sample and bare sample were immersed in phosphate-buffered saline (PBS) solution for 24 hours, after which the extract was collected and evaluated for bacterial studies. Gram-negative *Escherichia coli* (ATCC 25922) and Gram-positive *Staphylococcus aureus* (ATCC 25923) were studied as the bacterial types in this investigation. A frozen stock culture was used to extract the bacteria, which were then transferred to Mueller Hinton agar (MHA). The plates were incubated for 18 to 24 hours at 37°C. The bacteria were then transferred to 50 ml of sterile MHA and left to develop for 18 to 24 hours at 37°C. The bacterial strains were sub-cultured into new MHA in a ratio of 1:50 before inoculation and then were incubated for 2 hours at 37°C and 80 rpm.

Hemocompatibility Studies

Using the known procedure, a hemolytic test was performed. A 9:1 ratio of human vein blood (drawn from healthy donors) and trisodium citrate (3.2%) as the anticoagulant was used, and it was washed three times using PBS to collect erythrocytes (RBCs). The samples were incubated in 950 µl PBS at 37°C. Then, 50 µl of RBCs were added to the standard microcentrifuge tube, which was subsequently incubated at 37°C. After incubation, the tubes were centrifuged at 5000 rpm for 20 minutes. The negative control was PBS and the positive control was double distilled water (DDH_2_O) with RBCs and 0.1% sodium carbonate.

## Results

Surface morphology studies

**Figure 1:**
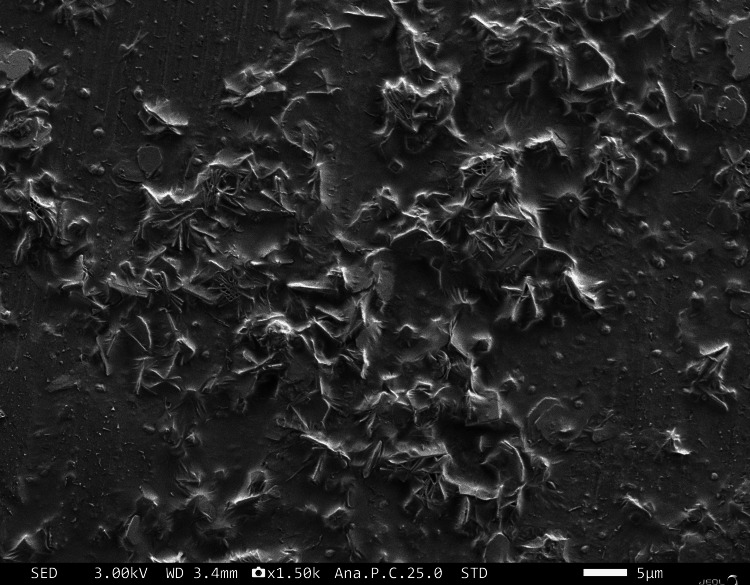
SEM image of Se/HA-coated Ti surface SEM: Scanning electron microscopy; Se: Selenium; HA: Hyaluronic acid; Ti: Titanium

The surface morphological study of the sol-gel coated Ti implant is shown in Figure 1. The surface properties display microneedles with plate-like morphology and small grain particles indicative of the heterogeneous coating on the entire surface. The surface properties are pivotal in the osseointegration process. The comparable results of Pajor K et al. had a synthesis of the selenium-hydroxyapatite forming a microstructure with nanoparticles on the surface [12]. The surface tendency of the Se/HA composite enhances cell adhesion and proliferation [13].

FTIR studies

**Figure 2:**
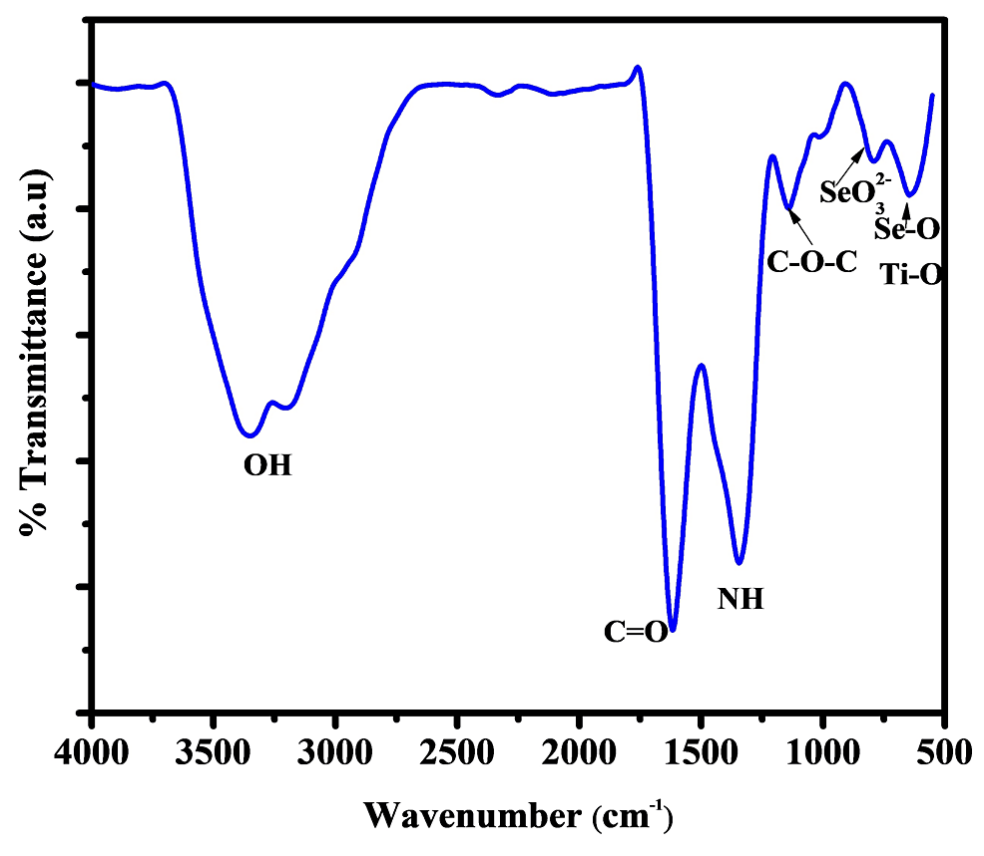
FTIR spectra of Se/HA-coated Ti surface FTIR: Fourier Transform Infrared; Se: Selenium; HA: Hyaluronic acid; Ti: Titanium; OH: Hydroxyl; C=O: Carboxyl; NH: Amide; C-O-C: Carbonyl; (SeO3)2-: Selenite ion; Ti-O: Titanium oxide ; Se-O: Selenium oxide

Figure 2 shows the FTIR spectra of Se/HA-coated Ti to establish the occurrence of functional groups. A strong broadband appeared for the hydroxyl group (-OH) at the wave number from 3600 to 3300 cm^-1^. The band 1620 cm^-1^ denotes the carboxyl group (C=O) asymmetric and symmetric vibration. The peak at 1340 cm^-1^ can be attributed to the amide I, II, and III groups [14]. The carbonyl group (C-O-C) peak is presented at 1137 cm^-1^. The minor peak at 800 cm^-1^ indicates selenite ions (SeO_3_^2-^). The strong metal oxide vibration peak at 640 cm^-1^ is characteristic of titanium oxide (Ti-O) and selenium oxide (Se-O) [15]. 

Contact angle measurements

**Figure 3:**
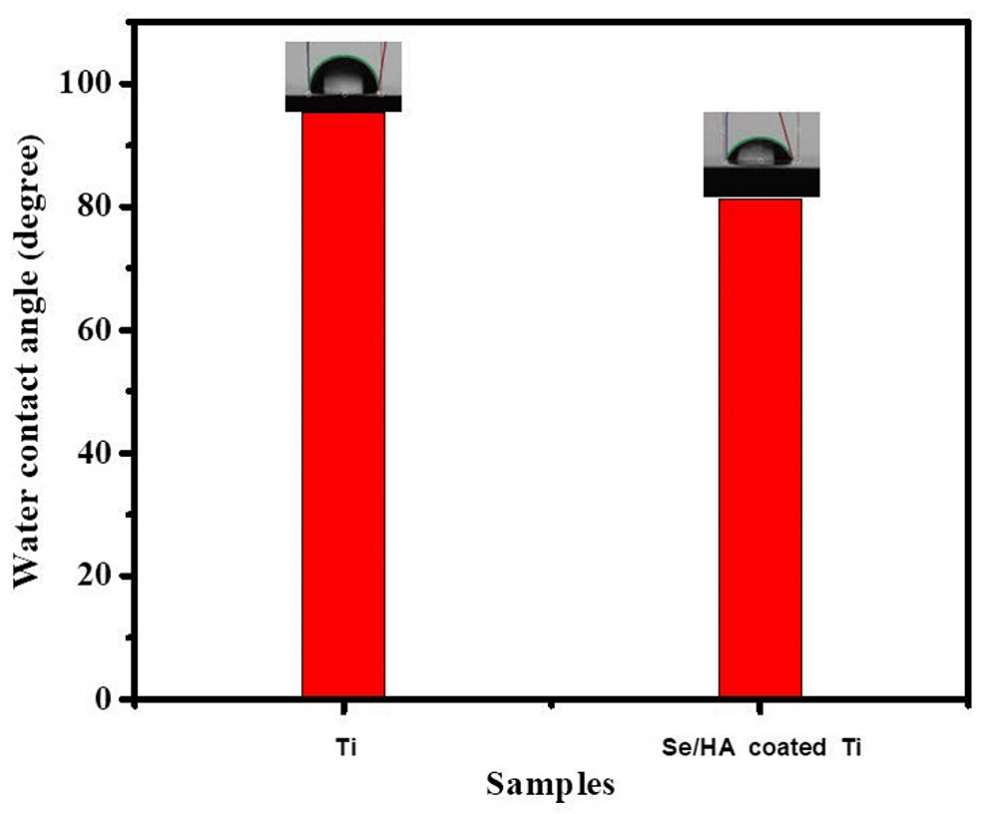
Water contact angle measurements of bare and coated Ti samples Ti: Titanium; Se: Selenium; HA: Hyaluronic acid

Surface wettability properties were assessed using contact angle measurement to determine the surface chemistry. Hydrophilic or hydrophobic characteristic features are displayed in Figure 3. The water contact angle of bare Ti and Se/HA-coated Ti substrates was observed: bare Ti showed 95.2° and Se/HA-coated Ti showed 81.3°. The coated sample's water contact angle is significantly lower than the bare sample. The surface wettability of the bare sample characterizes a hydrophobic surface, whereas the coating demonstrates a hydrophilic surface. The surface adsorption of coated Ti was high compared to bare Ti. The physicochemical properties of the surface phenomena play an important role in coated Ti, enhancing the implant's cell adhesion [16]. Due to physical signals, it is anticipated that the coating's enhanced surface roughness may improve cell adherence and proliferation. Hence, the Se/HA-coated Ti exhibited enhanced cell proliferation and cell adhesion.

Potentiodynamic polarization studies

**Figure 4:**
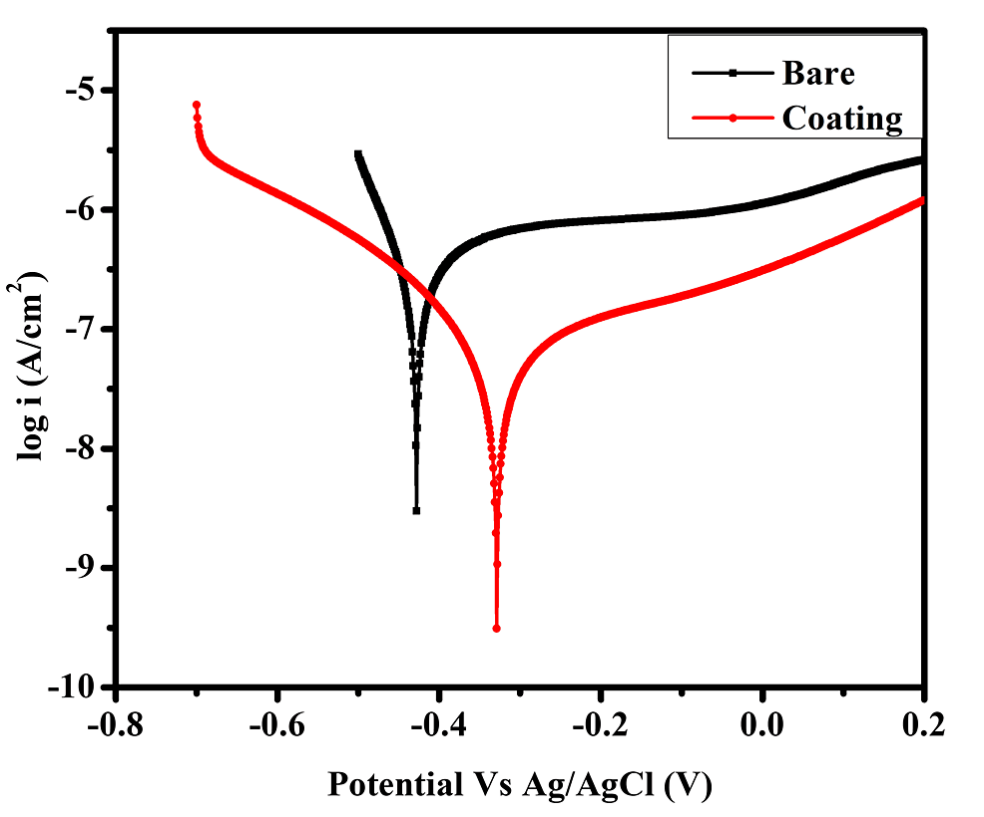
Potentiodynamic polarization studies of bare and coated Ti samples evaluated in SBF solutions Ti: Titanium; SBF: Simulated body fluid

To assess the corrosion protection of bare and Se/HA-coated Ti samples, evaluation was performed using simulated body fluid (SBF) solutions; the result is displayed in Figure 4. The evaluation of corrosion rate and polarization resistance is given in Table 1. From the potentiodynamic polarization studies, it was observed that the bare and coated samples exhibit Ecorr values of -0.45 V and -0.34 V, respectively. The deposition of the hybrid coating on Ti metal offers an icorr value of 6.48x10-8 A/cm2. The corrosion rate was reduced while the polarization resistance was increased due to the Se/HA coating, which although being a passive layer on the surface enhanced the corrosion resistance. The coating effectively reduced the corrosion rate because the formation of an anodic coating on the Ti surface hindered the penetration of corrosive ions from the body fluid solutions. A bare sample polarization was referred from Kalaiyarasan M *et al.* [17].

**Table 1:** Polarization resistance and corrosion rate of bare and coated samples evaluated in SBF solution Ti: Titanium; SBF: Simulated body fluid

Samples	E_corr_ (V)	i_corr_ (A/cm^2^)	Polarization resistance R_p_ (Ω)	Corrosion rate (mm/year x 10^-3^)
Bare Ti	-0.456	2.38x10^-7^	76650	1.567
Selenium/hyaluronic acid-coated Ti	-0.346	6.48x10^-8^	642276	0.052

Antibacterial studies

**Figure 5:**
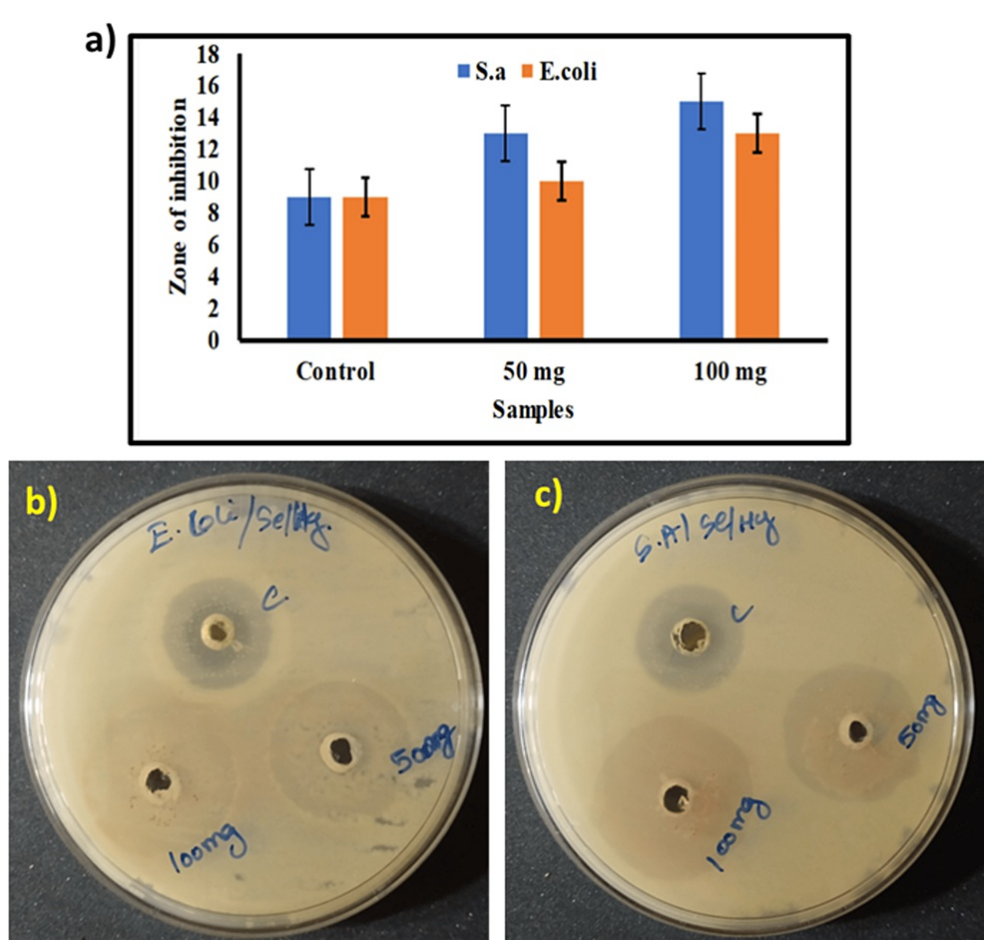
(a) Graph showing the percentage of zone of inhibition produced by coated Ti sample extract against (b) E. coli and (c) S. aureus for different concentrations Ti: Titanium; S.a: Staphylococcus aureus; E. coli: Escherichia coli

The antibacterial activity of Se/HA-coated Ti samples immersed for 24 hours in PBS solution is depicted as a bar graph (Figure 5a) and the zone of inhibition plates are shown in Figures 5b-5c. The zone of inhibition was evaluated with strains of *S. aureus* (gram-positive bacteria) and *E. coli* (gram-negative bacteria) at different concentrations. Antibiotic tetracycline was taken as control. The coated sample solution was seeded in concentrations of 50 mg and 100 mg of gram-positive and gram-negative bacteria each. As a result, a comparatively higher zone was found in 100 mg of gram-positive bacteria, which showcases the material potentiality against *S. aureus*, representing antibacterial properties. The composite coating of Se/HA killed the bacterial cell wall membrane of gram-positive bacteria due to bacteriostatic action. Additionally, 100 mg shows higher zone activity for gram-negative and gram-positive bacteria, due to the bacteriostatic activity of Se/HA composite material [18]. Improved antimicrobial properties of the Se/HA-coated Ti implant would be effective in implant applications.

Hemocompatibility studies

**Figure 6:**
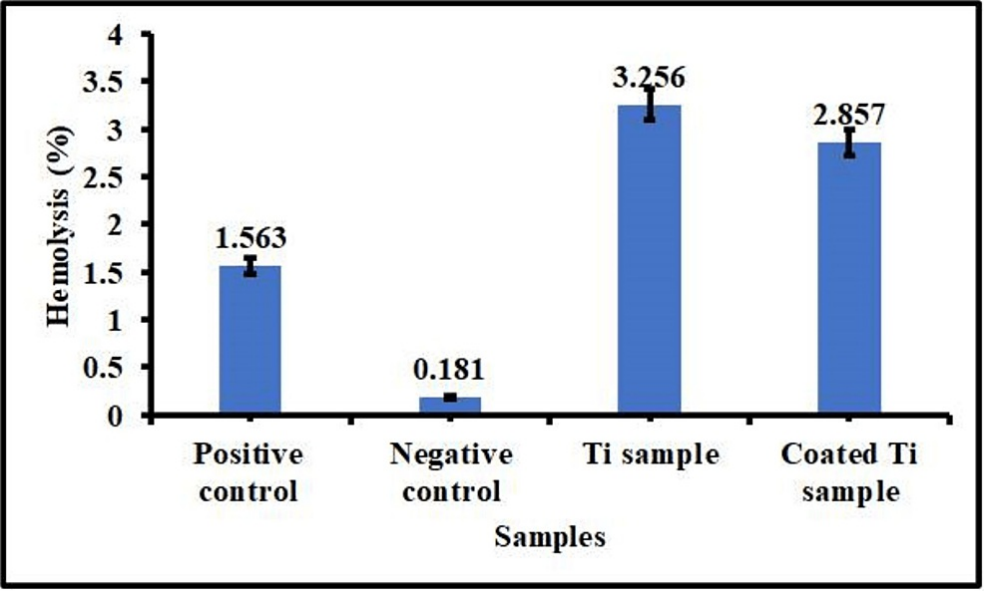
Hemocompatibility analysis of bare and coated Ti samples Ti: Titanium

Hemolysis is an important parameter for analyzing material compatibility and its harmful effects on RBCs. The implant material interacts with the blood tissues to cause lysis during the first implantation stage. Hence, an investigation of bare Ti and Se/HA-coated Ti hemolytic rate was performed and is given in Figure 6. The bare Ti metal revealed RBC damage of 3.25%, whereas coated Ti showed a significant reduction in the lysis rate by 2.85%. According to the ASTM (American Society for Testing and Materials) standard F756, implant material less than 5% is slightly hemolytic, and below 3% is non-hemolytic. According to the ASTM standard, the coated Ti implant shows a non-hemolytic nature [19,20]. The material shows compatible features with erythrocytes. 

## Discussion

The sol-gel coating of Se/HA-coated CP-Ti was successfully fabricated and a similar coating has been used for implant applications. The literature reports that few articles have been published on Se and composite coating on Ti for orthopedic applications. Based on the literature reports, Aksakal B *et al.* [20] studied the electrophoretic deposition of selenium in 316L SS, which shows a thick agglomerate coating on the surface. Comparatively, our results showed similar surface morphology, and a microneedle-like structure was observed (Figure 1), which might be due to the sol-like solution deposit on the Ti surface. Enhanced corrosion resistance was observed for the coated sample when compared to uncoated samples. The composite coating showed the noble direction shift of E_corr_ in a positive direction. The polarization resistance was increased due to the coating offering a passive layer to protect the Ti surface [16].

The main reason for an implant failure is a bacterial infection. To combat bacterial infection, antimicrobial properties are an essential physical characteristic of the implant material. In this study, Se/HA coating was evaluated, and it showed a higher zone of inhibition when tested with *S. aureus* (gram-positive bacteria). It was observed that the Se-incorporated hydroxyapatite material showed a lower zone of inhibition and higher cell interaction activity [21]. The formulation of HA-loaded antibiotic-based hydrogel coated on Ti-6Al-4V demonstrated outstanding multifunctional antibacterial activity for implant materials [22]. Li F *et al.* studied a one-step antimicrobial coating for Se nanoparticles (NPs), and they indicated that releasing Se NPs does not exhibit significant toxicity towards mammalian cells over 24 hours and that the Se NPs exhibited much lower cytotoxicity compared to silver (Ag) NPs. They are currently employed in some antimicrobial coatings for medical devices, which is significant evidence of the coatings' biocompatibility [23]. In our study, Se/HA-coated CP-Ti was able to kill the microbial infection on the implant site and also enhance the osseointegration process on the implant and bone tissue. Even though Se/HA-coated Ti upregulates the osteointegration and the microbial colonization; however, mechanically handling the coating Ti samples may cause the coating to peel off.

## Conclusions

In the present work, we fabricated a biocompatible coating of Se and HA and analyzed the antibacterial effect of the implant. The surface morphology of the coated sample exhibits microneedles with plate-like morphology, and the functional group was established by FTIR spectra. Potentiodynamic polarization studies revealed that the coated samples have superior corrosion resistance than the bare Ti. The antibacterial activity was evaluated using a zone of inhibition test, which showed that *S. aureus* has a higher zone in 100 mg concentration. The hemocompatibility assay of the material suggested a non-hemolytic nature. Overall, the coating offered better resistance and good antibacterial activity. Hence, the Se/HA-coated Ti implant is a good choice of material for clinical applications.
